# Multiple insecticide resistance and *Plasmodium* infection in the principal malaria vectors *Anopheles funestus* and *Anopheles gambiae* in a forested locality close to the Yaoundé airport, Cameroon

**DOI:** 10.12688/wellcomeopenres.15818.2

**Published:** 2020-11-05

**Authors:** Francis N. Nkemngo, Leon M. J. Mugenzi, Ebai Terence, Abdoulaye Niang, Murielle J. Wondji, Micareme Tchoupo, Nguiffo D. Nguete, Williams Tchapga, Helen Irving, Jacques D. M. Ntabi, Romuald Agonhossou, Terence S. Boussougou-Sambe, Romaric B. Akoton, Felix Koukouikila-Koussounda, Yudi T. Pinilla, Francine Ntoumi, Luc S. Djogbenou, Stephen M. Ghogomu, Cyrille Ndo, Ayola A. Adegnika, Steffen Borrmann, Charles S. Wondji

**Affiliations:** 1 Department of Parasitology and Medical Entomology, Centre for Research in Infectious Diseases (CRID), Yaounde, Centre Region, 237, Cameroon; 2 Department of Microbiology and Parasitology, University of Buea, Buea, South West, 237, Cameroon; 3 Department of Biochemistry and Molecular Biology, University of Buea, Buea, South West, 237, Cameroon; 4 Institute of Biodiversity Animal Health and Comparative Medicine, University of Glasgow, Glasgow, UK; 5 Department of Vector Biology, Liverpool School of Tropical Medicine, Liverpool, UK; 6 Fondation Congolaise pour la Recherche Medicale (FCRM), Brazzaville, Congo; 7 Université Marien Ngouabi, Brazzaville, Congo; 8 Institut Régional de Santé Publique, Université d'Abomey-Calavi, Cotonou, Benin; 9 Centre de Recherches Médicales de Lambaréné, Lambaréné, Gabon; 10 Institute for Tropical Medicine, University of Tübingen, Tübingen, Germany; 11 Department of Biological Sciences, Faculty of Medicine and Pharmaceutical Sciences, University of Douala, Douala, Cameroon; 12 Eberhard Karls Universität Tübingen,, Tübingen, Germany; 13 German Center for Infection Research (DZIF), Tübingen, Germany

**Keywords:** Malaria, Anopheles funestus, Anopheles gambiae, Plasmodium infection, Insecticide resistance, Vector control, Cameroon

## Abstract

**Background:** Reducing the burden of malaria requires better understanding of vector populations, particularly in forested regions where the incidence remains elevated. Here, we characterized malaria vectors in a locality near the Yaoundé international airport, Cameroon, including species composition, abundance, *Plasmodium* infection rate, insecticide resistance profiles and underlying resistance mechanisms.

**Methods:** Blood-fed adult mosquitoes resting indoors were aspirated from houses in April 2019 at Elende, a locality situated 2 km from the Yaoundé-Nsimalen airport. Female mosquitoes were forced to lay eggs to generate F _1_ adults. Bioassays were performed to assess resistance profile to the four insecticides classes. The threshold of insecticide susceptibility was defined above 98% mortality rate and mortality rates below 90% were indicative of confirmed insecticide resistance. Furthermore, the molecular basis of resistance and *Plasmodium *infection rates were investigated.

**Results:** *Anopheles funestus *s.s. was the most abundant species in Elende (85%) followed by *Anopheles gambiae *s.s. (15%) with both having similar sporozoite rate. Both species exhibited high levels of resistance to the pyrethroids, permethrin and deltamethrin (<40% mortality). *An. gambiae* s.s. was resistant to DDT (9.9% mortality) and bendiocarb (54% mortality) while susceptible to organophosphate. *An. funestus* s.s. was resistant to dieldrin (1% mortality), DDT (86% mortality) but susceptible to carbamates and organophosphates. The L119F-GSTe2 resistance allele (8%) and G119S *ace*-1 resistance allele (15%) were detected in *An. funestus *s.s. and *An. gambiae *s.s., respectively *.* Furthermore, the high pyrethroid/DDT resistances in *An. gambiae* corresponded with an increase frequency of 1014F *kdr* allele (95%). Transcriptional profiling of candidate cytochrome P450 genes reveals the over-expression of *CYP6P5*, *CYP6P9a* and *CYP6P9b.*

**Conclusion:** The resistance to multiple insecticide classes observed in these vector populations alongside the significant *Plasmodium *sporozoite rate highlights the challenges that vector control programs encounter in sustaining the regular benefits of contemporary insecticide-based control interventions in forested areas.

## Abbreviations

DDT: dichlorodiethyltrichloroethane; DNA: deoxyribonucleic acid; dNTPs: deoxyribonucleoside triphosphates; GSTe2: glutathione S-transferase epsilon 2; IRS: indoor residual spraying; kdr: knockdown resistance mutation; LLIN: long-lasting insecticidal net; NMCP: National Malaria Control Programme; PBO: piperonyl butoxide; PCR: polymerase chain reaction; *s.l.*: *sensu lato*; s.s.: *sensu stricto;* WHO: World Health Organization.

## Introduction

Malaria is the major vector-borne disease globally and a leading public health problem ^1^. In 2018, there were roughly 228 million cases of the disease and about 405,000 malaria-related deaths. Approximately 67% of deaths recorded were children aged below five years ^1^. Although, a shift in focus from malaria control to elimination was declared by the WHO in 2012, it was observed that between 2015 and 2018; no considerable progress was achieved in decreasing global malaria cases. Rather, there was a reported increase in malaria victims in 2018 compared with the previous years in ten African countries scoring the highest burden of the disease ^1^.

To this effect, the WHO Global Technical Strategy for Malaria (2016–2030) outlines a pathway for malaria control and elimination and designates a target for a 90% reduction in global malaria mortality rates by 2030 relative to a 2015 baseline ^2^. In this vein, the recent certification of Algeria and Argentina as malaria-free countries by the World Health Organization (WHO) has been a historic achievement for universal health coverage, and serving as a model in demonstrating the feasibility of malaria elimination in the Afro-tropical region ^1^. This success was in part largely attributed to a coordinated system of vector control interventions such as long-lasting insecticidal nets (LLINs) and indoor residual spraying (IRS) among others, including prompt diagnosis, effective treatment and efficient surveillance response system ^3^. Despite this, the efficacy of these insecticide based-vector control tools is compromised by the growing problem of insecticide resistance widely exhibited by *Anopheles* vectors across the African continent ^4^.

In Cameroon, malaria is endemic, with the entire population considered to be at risk. In 2018, the country accounted for 3% (6,840,000) of malaria cases and recorded about 3,000 deaths within the WHO African region ^1^. In order to reduce the malaria burden, the Cameroonian government supports the National Malaria Control Program (NMCP) and other partners who have established a strategic plan to achieve the goal of ensuring equal access to quality and affordable tools necessary for sustaining malaria control and elimination. The malaria interventions include mass distribution of LLINs, prompt and effective diagnosis, artemisinin-based combination therapy (ACTs), seasonal malaria chemoprevention (SMC) and intermittent preventive therapy (IPT) in pregnant women through administration of sulfadoxine-pyrimethamine ^5^. Moreover, to further strengthen the current vector control intervention, an IRS campaign is to be launched in Cameroon for the first time by the United States President Malaria Initiative (PMI) project. Unfortunately, the wide spread nature of insecticide resistance in malaria vectors to common insecticides used to impregnate nets and spray walls in Cameroon ^5– 10^ threatens the success of this strategy.

In Cameroon, malaria is mainly transmitted by *An. gambiae s.s, An. coluzzii, An. arabiensis, An. funestus* s.s. *, An. nili* and *An. moucheti vectors* ^5^ which have different geographical distributions across the country. However, despite such an important epidemiological role played by these vectors, data on the pattern of malaria transmission across different ecological settings in Cameroon including urban, peri-urban and rural remains insufficient ^11^. This represents a major challenge for the realization of effective universal coverage of LLINs and universal access to anti-malaria drugs and treatment since malaria control requires a good understanding of the transmission dynamics. Currently, the absence of sufficient information on vector bionomics and disease transmission in peri-urban areas could constitute a potentially steady malaria pool. This may serve as a bridge between rural and urban regions especially if high or residual transmission are maintained in such areas ^12^ thus posing a massive challenge for malaria control.

In order to facilitate and reinforce the National Malaria Control Program in their efforts to implementing sustainable and efficacious vector control interventions, this study investigated the entomological component of malaria transmission in a peri-urban setting within the forested region of Cameroon. This included the characterization of the endophilic mosquito species composition, investigation of the insecticide resistance profiles of *An. funestus* s.s. and *An. gambiae* s.s. populations collected in Elende in 2019, a forested peri-urban area located 2 km away from the Nsimalen International Airport of Yaoundé, the capital city of Cameroon.

## Methods

### Study area and mosquito collection

**Figure 1.**
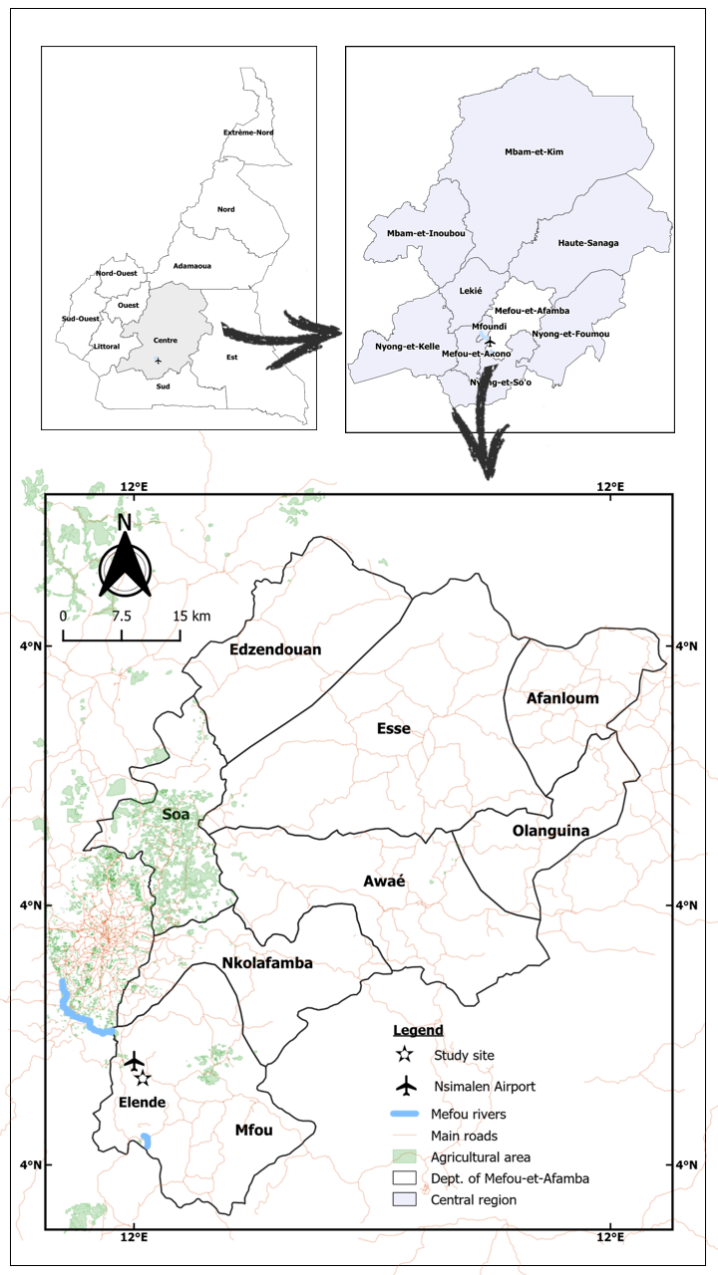
Map of Elende study area; showing its close proximity to the Nsimalen- International Airport of Yaoundé, capital city of Cameroon.

Blood fed female mosquitoes were collected indoor in April 2019 from Elende (3°41'57.27''N, 11°33'28.46''E) ( Figure 1), district of Nkolmefou I, a peri-urban locality close to Yaoundé; the capital city of Cameroon. This area is about 2 km away from the Nsimalen International Airport and close to the Mefou River. This locality is categorized by an equatorial Guinean climate, represented by two rainy seasons (August-October and March-May) and two dry seasons (November-February and June–July). The yearly average rainfall is 1800 mm while mean annual temperatures range between 19–28°C, and the mean humidity varies between 65–80%. The vegetation around the village is predominantly made up of an equatorial forest which is being degraded for farming activities and infrastructure. Road construction activities and deforestation are ongoing in this locality, an environmental modification system which creates temporal and permanent breeding sites for malaria vectors such as *An. gambiae* s.s. Moreover, the village is proximal to marshy lands and streams joining major rivers as these persistent water masses are ideal conditions, particularly favoring vector multiplication of *An. funestus* s.s. mosquitoes. Subsistence farming including cassava cultivation and vegetable cropping (particularly tomatoes, pepper, lettuce and watermelon) are the main human activities in this locality. The yield from this farming practices are greatly enhanced by the intensive use of pesticides. Also, household animals such as cattle, goats and sheep are present on a minor scale. Furthermore, several fish ponds bordered with vegetation exist in this village. This might encourage the development and growth of immature stages of the species within the *An. funestus* group. In this locality, pyrethroids containing LLINs (PermaNet 2.0) is the main prevention method with a coverage of around 60% ^5^ as the area is endemic for malaria.

Following verbal approval from the chief and household heads; blood-fed indoor resting adult mosquitoes (F0s) were collected between 06.00am and 11.00am from 15–20 randomly selected houses to avoid sampling individuals from single female egg batches and to obtain a representative population-level data for this area. The Prokopack electrical aspirator (John W Hock Co, Gainesville, FL, USA) was used for mosquito collection, after which they were kept in a humid cage and later transported to the insectarium of the Centre for Research in Infectious Diseases (CRID), Yaoundé. Field collected females were placed in a cage to rest for 1 hour prior to morphological identification ^13^. Each live mosquito sample was aspirated from the cage in to a hemolysis tube and observed microscopically for distinct morphological differences of wings, mouthparts and size based on the Afro-tropical anopheline key ^13^. Specimens of the *An. funestus* group and *An. gambiae* complex were then placed in two separate small-sized labeled cages and left for 4–5 days feeding on 10% sugar soaked in cotton wool for them to become fully gravid. A forced-egg laying method as previously described ^14, 15^ was utilized for individual oviposition of females. After oviposition, all the carcasses of the F _0_ were kept in separate 1.5-ml tubes containing silica gel and stored at -20°C prior to molecular analysis.

### Molecular species identification

The Livak method was used to extract genomic DNA from the head/thorax of each individually oviposited and non-oviposited field-caught female (F _0_s) ^16^. DNA extracts were quantified using NanoDrop™ spectrophotometer (Thermo Scientific, Wilmington, USA). A SINE-200 PCR ^17^ and cocktail PCR ^18^ were performed to identify the different species within the *An. gambiae* s.l. complex and *An. funestus* s.l. group, respectively. The ribosomal DNA internal transcribed spacer region 2 (ITS2) was amplified to identify the undetermined species using the protocol of Hackett *et al.* ^19^. The PCR amplicon of 840 bp was purified with Exonuclease I (Exo I) and Shrimp Alkaline Phosphate method (Exo-SAP) based on the New England Biolabs procedure (NEB, MA, USA) and directly sequenced commercially.

### *Plasmodium* infection rates

*Plasmodium* infection was assessed in 150 *An. funestus* s.s. and 39 *An. gambiae* s.s. F0 females. Genomic DNA was extracted from the head and thorax of each specimen, and infection with *P. falciparum* or OVM+ ( *P. ovale, P. vivax* and *P. malariae*) was detected using the TaqMan assay as described previously with slight modifications (1 cycle at 95°C/10mins and 40 cycles at 92°C/15s and 60°C/1min) ^20^. Sequentially, nested PCR was conducted on all the positive samples to confirm the TaqMan assay results and to specifically differentiate between the OVM species obtained ^21^.

### WHO insecticide susceptibility bioassays

Various insecticides employed in control of malaria vectors were tested in bioassays to assess the resistance profile of the *An. funestus* s.s. and *An. gambiae* s.s. mosquito populations, according to the WHO protocol ^22^. Brand new insecticide-impregnated papers were supplied by WHO reference center (Vector Control Research Unit, University Sains Malaysia, Penang, Malaysia). Two- to five-day-old, unfed F _1_ female *An. funestus* s.s. were exposed for 1 h to discriminating concentrations of the following insecticides: pyrethroids [the class I pyrethroid permethrin (0.75%); n=80, and the class II pyrethroid deltamethrin (0.05%); n=78]; the organochlorines [DDT (4%); n=80 and dieldrin (4%); n=95]; the carbamates [bendiocarb (0.1%); n=79 and propoxur (0.1%); n=87]; and the organophosphate malathion (5%) (n=99). The remaining population constituted the control group (n=40). Similarly, *An. gambiae* s.s. female mosquitoes were exposed to all the insecticides [permethrin (n=86), deltamethrin (n=89), DDT (n=90), bendiocarb (n=87), malathion (n=84)] except dieldrin and propoxur; instead, fenithrothion (5%) (n=84) and pirimiphos-methyl (1.25%) (n=61) were added.

Furthermore, in order to establish the resistance intensity to pyrethroid insecticides, the *An. gambiae* s.s. F _1_ generations from Elende were tested on permethrin (n=171) and deltamethrin (n=167) concentrations of 5x and 10x for 60 mins. Mortality rates were recorded 24 h post exposure. The dead mosquitoes were kept in 1.5-ml tubes containing silica gel while the survivors were placed in tubes containing RNALater and stored at -80°C for molecular analyses.

A set of 20-25 mosquitoes exposed to untreated papers were used as control for each test. The experiment was carried out at ambient temperatures of 25°C ± 2°C and 80% ± 10% relative humidity. A mortality rate >98% of the mosquito populations was considered susceptible to the insecticide, meanwhile suspected resistance was considered at mortality between 90–98%, and resistant where mortality was found to be <90%.

### Piperonyl butoxide (PBO) synergist assay

Only females of *An*. *funestus* s.s. were used for this assay since *An*. *gambiae* s.s. was inadequate in the study area at the period of collection. In order to determine the possible implication of cytochrome P450s in the observed phenotypic resistance to pyrethroids, two to five days old F _1_ female *An. funestus* s.s. were initially exposed to 4% PBO for 1 h proceeded by immediate second exposure to permethrin (0.75%) or deltamethrin (0.05%) for another 1 h exposure. The mortality was determined at 24 h post exposure and compared with mortality achieved for mosquitoes subjected to the pyrethroids only. Differences in mortality among the various groups were analyzed and recorded ^22^.

### Assessment of bed net efficacy using cone assay

Due to the low abundance of *Anopheles gambiae* s.s., in this locality, we examined the efficacy of bed nets approved by WHO against the Elende *An*. *funestus* s.s. population. This was done to evaluate the impact of resistance on insecticide targeted interventions against *Anopheles* vectors in this village. Cone bioassays were performed according to the WHO procedure ^23^ using four standard types of LLINs (Olyset Plus, Olyset Net, PermaNet 2.0 and PermaNet 3.0 (side and roof)). Brand new PermaNet and OlysetNet nets manufactured in 2018 were supplied by the companies, Vestergaard (Lausanne, Switzerland) and Sumitomo Chemical Plc (London, UK), respectively. Four replicates of ten unfed mosquitoes were introduced into each plastic cone attached on pieces of fresh, unused bed nets of dimension 30 cm × 30 cm: Olyset®Net (containing 2% permethrin), OlysetPlus®Net (containing 2% permethrin combined with 1% of the synergist PBO), PermaNet®2.0 (containing 1.4–1.8 g/kg ± 25% deltamethrin), and PermaNet®3.0 [both the side panel (containing 2.1–2.8 g/ kg ± 25% deltamethrin) and the roof (containing 4.0 g/kg ± 25% deltamethrin, combined with 25 g/kg ± 25% of PBO)]. In a similar way, four replicates of ten mosquitoes each were included in each batch of the LLIN cone test and exposed to an untreated net to serve as negative control. For each test, 3 min was the exposure time. After exposure, the mosquitoes were gently and immediately removed from the cones using a mouth aspirator, transferred into paper cups and fed with 10% sucrose soaked in cotton wool. The number of mosquitoes knocked-down was recorded after 1 h while mortality was calculated after 24 h of observation. The experiment was carried out at ambient temperature of 25°C ± 2°C and 80% ± 10% relative humidity.

### Genotyping of L119F-GSTe2, *CYP6P9a*, A296S-RDL, resistance marker in *Anopheles funestus s.s*

An allele-specific PCR was used to genotype and determine the frequency of the L119F-GSTe2 mutation in *An. funestus* s.s. F _0_ mosquito population of Elende as previously described ^24, 25^. This was to investigate the role of glutathione S-transferases in DDT resistance. The presence of *CYP6P9a* resistance allele associated with resistance to pyrethroids was genotyped by a PCR-RFLP assay ^26^ while the A296S-RDL mutation known to be linked with dieldrin resistance was also genotyped by TaqMan assay ^27^.

### Genotyping of L1014F, L1014S *kdr* and G119S *ace-1* resistance marker in *Anopheles gambiae* s.s.

The L1014F-kdr and L1014S-kdr mutations involved in pyrethroid and DDT resistance in *An. gambiae* s.s. were genotyped in F _0_ Elende mosquitoes using the TaqMan assay ^28^. In addition, the G119S *ace-1* responsible for carbamate and organophosphate resistance in *An. gambiae* s.s. was also genotyped in Elende mosquitoes using a TaqMan assay as previously described ^29^.

### Transcription profiling of candidate resistance associated genes

A quantitative reverse transcriptase PCR (qRT-PCR) was done to investigate the prominent role of some previously reported Cytochrome P450 detoxification genes ( *CYP325A*, *CYP6P5*, *CYP6P9a* and *CYP6P9b*) in *An. funestus* s.s. associated to the phenotypic resistance recorded during bioassay ^30– 32^. Using a triplicate of 10 F _1_ females each that recovered after 1 h exposure to permethrin from Elende and 3 batches of unexposed 10 F _1_ females that were used as control samples; total RNA extraction, cDNA synthesis and qRT-PCR reactions were performed as earlier reported ^30^. Fold change and expression of each gene in resistant (R) and control (C) samples were computed according to 2-ΔΔCq method ^33^ following standardization with housekeeping gene: ribosomal protein S7 (RSP7) (AFUN007153-RA) and the Actin 5C (AFUN006819) genes.

An earlier version of this article can be found on Research Square (DOI: https://doi.org/10.21203/rs.2.23277/v1).

## Results

### Species identification

A total of 269 adult resting female *Anopheles* mosquitoes were collected indoors at Elende over a two-day period. Out of the total sample collected; 230 (85.50%) were members of *An. funestus* group while the remaining 39 (14.49%) species belonged to the *An. gambiae* complex.

From the 120 *An. funestus* s.l. mosquitoes that were chosen randomly and identified using cocktail PCR, *An. funestus* s.s. was dominant [98.34% (118/120)] with *An. rivolurum* [0.83% (01/120)] and *An. vaneedeni* [0.83% (01/120)] also detected. Similarly, from the 39 *An. gambiae s.l.* mosquitoes collected and analyzed using SINE PCR, all were *An. gambiae* s.s. Furthermore, the results of ITS-2 sequencing confirmed the undetermined mosquitoes from cocktail PCR to be *An. funestus* s.s [GenBank accession numbers: MT991011 to MT991016]. Out of the 230 and 39 mosquitoes morphologically identified, 196 (85.22%) *An. funestus* s.s. and 20 (51.28%) *An. gambiae* s.s. laid eggs respectively by the forced egg laying technique (see *Underlying data* for raw PCR values ^34^).

### *Plasmodium* infection rate

The analysis of the head and thorax revealed 09/150 (6%, CI: 4.15-8.57) infected *An. funestus* s.s. mosquitoes, which included 7 (4.67%, CI: 2.35-7.16) *P. falciparum* and 2 (1.33%, CI: 0.66-2.97) mosquitoes infected with either *P. ovale*, *P. vivax* or *P. malariae* (OVM). The *Plasmodium* infected mosquitoes revealed by TaqMan were further tested with nested PCR. Results of nested PCR reported 5 *P. falciparum* positive samples and 2 samples infected with *P. malariae*.

Out of 39 collected *An. gambiae* s.s., 5.13% [(02/39) (CI: 2.57-7.13)] were infected with *P. falciparum* and none for *P. OVM* or mixed infections. Nested PCR confirmed 1 positive *P. falciparum* sample out of the 2 infected samples detected by TaqMan assay (see *Underlying data* for raw values ^34^).

### Insecticide susceptibility assays

**Figure 2.**
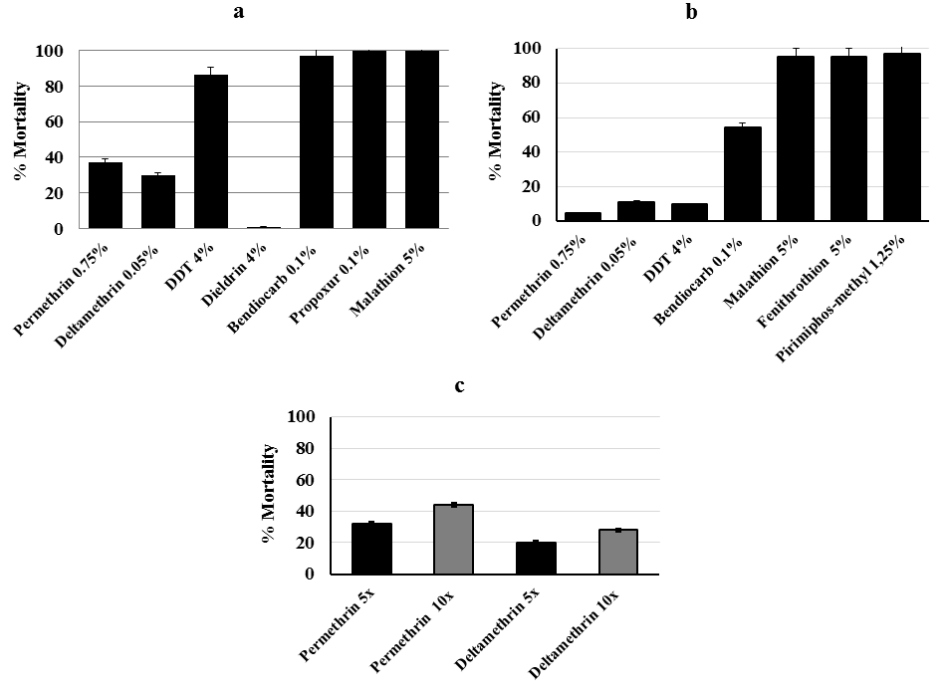
Results of WHO insecticides susceptibility test. ( **a**) Susceptibility profile of female *An. funestus* s.s. (F1 progeny; N=638) from Elende following exposure to various public health insecticides. ( **b**) Susceptibility profile of Elende female *An. gambaie* s.s. (F1 progeny; N=621). ( **c**) Resistance intensity of *An. gambiae* s.s. (F1; N=358) on exposure to 5x and 10x permethrin and deltamethrin each; N is the total number of mosquitoes tested. Error bars represent standard error of the mean.

A sum of 638 F _1_ female *An. funestus* s.s. mosquitoes were tested to determine the resistance profile to seven insecticides ( Figure 2). *An. funestus* s.s. was resistant to class I and class II pyrethroids. Mortality to permethrin (type I pyrethroid) was 37.00±6.59% while for deltamethrin (type II pyrethroid), mortality was 29.80±0.86% ( Figure 2a). Regarding the organochlorine insecticides, moderate resistance to DDT was recorded with a mortality rate of 86.25±8.98% ( Figure 2a). However, extremely high resistance was noticed for dieldrin with a recorded mortality value of 1.04±1.04% ( Figure 2a). For the carbamates, full susceptibility was observed for propoxur with a mortality rate of 100% whereas a near susceptibility was noted for bendiocarb with a mortality value of 97.37±2.63% ( Figure 2a). A full susceptibility was obtained for the organophosphate, malathion with 100% mortality ( Figure 2a).

A total of 979 female F1 *An. gambiae* s.s. progeny was tested to evaluate the resistance profile to seven insecticides as well as the resistance intensity to the pyrethroids. The *An. gambiae* s.s. population was highly resistant to class I and class II pyrethroids. Mortality to permethrin was 5.00±0.06% and 11.32±1.40% for deltamethrin ( Figure 2b). Due to the high resistance observed for the diagnostic concentration of pyrethroids, an increased concentration of permethrin (5x and 10x) and deltamethrin (5x and 10x) was used to further determine the extent of resistance intensity on the F _1_ *An. gambiae* s.s. population. High intensity resistance to 5x and 10x permethrin was observed with a recorded mortality rate of 31.74% and 44.30% respectively which according to the WHO guidelines ^22^ indicates a high intensity of resistance in Elende. In a similar manner, mortality rate upon exposure to 5x and 10x deltamethrin was observed at 19.80% and 29.00%, respectively indicating a high intensity resistant population ( Figure 2b). Regarding the organochloride insecticide DDT, an elevated resistance pattern was observed with a mortality rate of 9.98±1.03% ( Figure 2b). Resistance to the carbamate insecticide, bendiocarb was recorded with a mortality value of 54.56±1.05% ( Figure 2b). Moreover, for the organophosphate insecticides, mortality rates of 95.24±1.94%, 95.23±0.09%, and 96.75±1.63% were observed for malathion, fenithrothion and pirimiphos-methyl respectively ( Figure 2b) suggesting possible resistance that necessitates further confirmation (see *Underlying data* ^34^).

### PBO synergist assays with *An. funestus* s.s.

**Figure 3.**
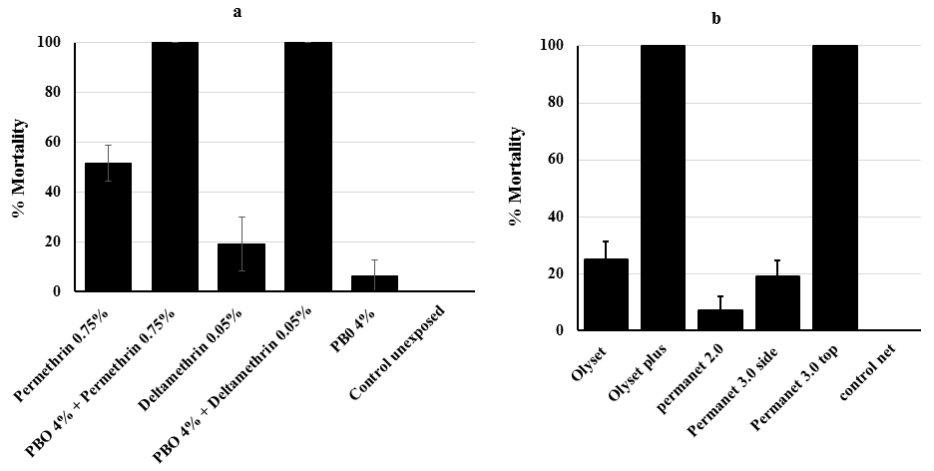
Susceptibility profile of *An. funestus* s.s. to synergist and cone assays from Elende. ( **a**) Activities of PBO synergist assay on *An. funestus* s.s. (F1 population; N=489). ( **b**) Recorded mortalities following 3-min exposure by cone assay of *An. funestus* s.s. (F1 generation; N=230) from Elende to Olyset, Olyset Plus, PermaNet 2.0, PermaNet 3.0 (side) and PermaNet 3.0 (roof); N is the total number of mosquitoes tested. Data are shown as mean±SEM.

A total of 489 female F1 progeny was employed for the PBO synergist assay. Initial exposure of 169 F _1_ *An. funestus* s.s. mosquitoes to PBO led to return of susceptibility to both class I and II pyrethroids with full mortality recorded after PBO + pyrethroid exposure from 51.50±7.19% to 100% for permethrin and from 19.22±10.76% to 100% mortality for deltamethrin ( Figure 3a) (see *Underlying data* ^34^). Briefly, the number of mosquitoes per treatment includes: permethrin (n=85), PBO + permethrin (n=80), deltamethrin (n=96), PBO + deltamethrin (n=89), PBO only (n=74) and control (n=65).

### Assessment of bed net efficacy on *An. funestus* s.s. population by cone assays

A total of 230 F1 female mosquitoes were used for the cone assay. Out of the 192 F1 *An. funestus* s.s. used to assess the efficacy of conventional bed nets, a reduced efficacy was seen for both pyrethroid-only impregnated nets with low mortality rate observed for Olyset net (24.97±6.45%) and PermaNet 2.0 (7.27±4.75%) 24 h after mosquito exposure ( Figure 3b). Conversely, nets impregnated with PBO exhibited a significantly greater efficacy with full susceptibility for both PermaNet 3.0 top (100% mortality) and Olyset plus (100% mortality). However, a lower efficacy was recorded for the side part of PermaNet 3.0 (19.17 ± 5.46% mortality). The susceptible strain for *An. funestus* FANG, used as control demonstrated full mortality to all nets ( Figure 3b) (see *Underlying data* ^34^).

### Genotyping of L119F GSTe2, Cyp6P9a and A296S-RDL resistance markers in *An. funestus* s.s.

**Figure 4.**
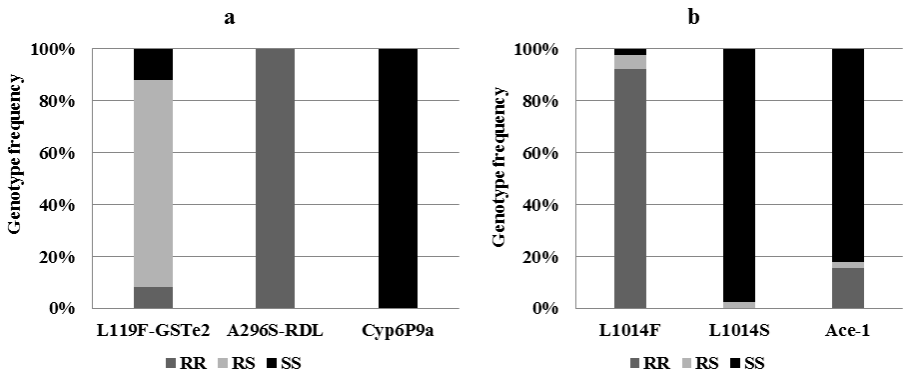
Temporal distribution of resistance markers in Elende. ( **a**) *An. funestus s.s*: Frequency of the L119F-GSTe2 conferring DDT resistance; A296S-RDL mutation conferring dieldrin resistance; Cyp6P9a related with pyrethroid resistance. ( **b**) *An. gambiae s.s*: Frequency of the L1014F conferring pyrethroids and DDT *kdr*W resistance; L1014S related with pyrethroids and DDT *kdr*E resistance; G119S *ace-1* conferring carbamate and organophosphate resistance. RR, homozygote for resistant allele; RS, heterozygote; SS, homozygote for susceptible allele.

A subset of 50 F _0_ females collected from the field was used for genotyping L119F-GSTe2 molecular marker ( Figure 4a). Out of this cohort, 04 mosquito samples were homozygous resistant (RR) (8%), 40 were heterozygous (RS) (80%) and 6 were homozygous susceptible (SS) (12%). Overall, the frequency of the 119F resistant allele (R) was 48% and 52% for the L119 susceptible allele (S).

The PCR-RFLP genotyping of *CYP6P9a* revealed that all the mosquitoes were homozygous susceptible with a band size of about 500 bp ( Figure 4a) indicating that this mutation is absent in *An. funestus* s.s. population of Elende.

Also, the 50 samples genotyped for A296S marker were all homozygous resistant RR (100%), revealing that the mutation is fixed in this population ( Figure 4a) in line with the high dieldrin resistance observed (see *Underlying data* ^34^).

### Genotyping of L1014F, L1014S *kdr* and G119S ace 1 markers in *An. gambiae* s.s.

Out of 39 samples genotyped for L1014F *kdr* resistance marker, 36 were homozygous resistant RR (92.30%), 02 were heterozygous RS (5.13%) and 01 homozygous susceptible SS (2.56%) with a 1014F resistant allele frequency of 94.86% ( Figure 4b). Likewise, out of the 39 samples genotyped for the L1014S marker, 01 was RS (2.56%) and 38 were SS (97.43%) ( Figure 4b). Thus, a very low frequency of 1.28% was observed for the1014S resistant allele.

Similarly, out of 39 samples genotyped for the G119S resistance marker, 06 were homozygous resistant RR (15.38%), 01 was heterozygous resistant RS (2.56%) and 32 were found to be homozygous susceptible SS (82.05%) ( Figure 4b). Overall, the frequency of the 119S resistant allele was 16.66% (see *Underlying data* ^34^).

### Transcriptional profiling of candidate genes

**Figure 5.**
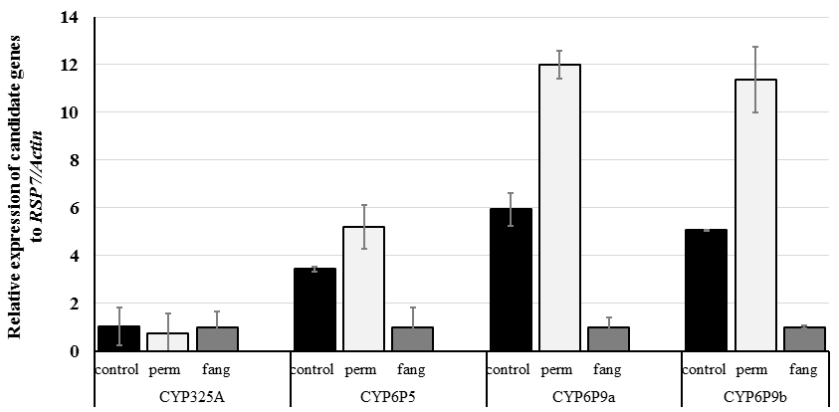
Differential expression by qRT-PCR of the major Cytochrome P450 genes ( *CYP*325A, *CYP*6P5, *CYP*6P9a and *CYP*6P9b) in *An. funestus* s.s. in Elende compared with the susceptible *A. funestus* s.s. strain FANG. Error bars represent standard errors of the mean.

 qRT-PCR was done to examine the role of some previously reported Cytochrome P450 metabolic genes in *An. funestus* s.s. linked to the resistance observed during bioassay (see *Underlying data* ^34^). The qRT-PCR results reveal that *CYP6P5*, *CYP6P9a* and *CYP6P9b* genes known to be involved in pyrethroid resistance are significantly up-regulated in *An. funestus* s.s. population from Elende as compared to the susceptible laboratory strain FANG ( Figure 5). Both *CYP6P9a* and *CYP6P9b* exhibited a 6-fold change in Elende resistant mosquito when compared to FANG (P<0.05) whereas *CYP6P5* displayed a 2.20-fold change difference in expression (P<0.05) between the wild mosquitoes and susceptible strain. On the other hand, when comparing permethrin exposed to the unexposed (control) mosquito and FANG strain, *CYP325A* was not significantly expressed.

## Discussion

Rapid scaling up of vector control interventions is ongoing in Cameroon, where malaria is highly endemic. As such, characterization of local vectors alongside investigation of their resistance profile is essential for the effective designing and execution of successful and sustainable vector control interventions as well as for evaluating the impact of insecticides resistance.

In the past, the possibility of generating a large number of F _1_ progeny from small numbers of field collected mosquito for molecular characterization constituted a major hindrance for colonizing *An. funestus* in the lab. However, this limitation has been resolved by the invention of the forced egg laying method ^14^. An easy approach to addressing this barrier is by collecting indoor resting blood fed female mosquitoes and putting each of them in a confined 1.5-ml tube to forcefully lay eggs. This method has made feasible the substantial evaluation of the susceptibility profile of this mosquito species population against different classes of insecticide. Nevertheless, for experiments involving F _1_ adults to be informative, it is cardinal that, the offspring obtained by this technique should not be bias and family isolation effects must be reduced such that the progeny are typical of the overall population. In this regard, pooled egg batches were reared together and the F _1_ adults were randomly combined in cages for the various assays.

With the possibility of generating a large number of progenies from field collected female *An. funestus* and *An. gambiae* mosquitoes, this study therefore characterized the principal malaria vectors in a peri-urban setting within the forested region of Cameroon, located 2 km away from the Yaoundé -Nsimalen International airport.

### Mosquito species composition in Elende

From the nine species of *An. funestus* s.l. group described, *An. funestus* s.s., *An. rivulorum* and *An. vaneedeni* were detected in Elende, with abundance of 98.34%, 0.83% and 0.83% respectively. This result is similar to a study conducted in Tibati ^35^ and Gounougou (northern region of Cameroon), where *An. funestus* s.s. accounted for 99.50% of the species collected and *Anopheles leesoni* was 0.50% ^9^. Likewise, the result reflects the species abundance in a published study conducted in Mibellon (Cameroon), where *An. funestus* s.s. was the only dominant vector found within the group ^11^. The superiority of *An. funestus* s.s. was also reported in Kpome-Benin (West Africa) ^36^. Since this study was done at a single point in time, we cannot exclude the presence of other Anopheline species. More so, the study may have limited the collection of outdoor resting members of the group, since mosquito sampling was concentrated indoors. However, this differs with the distribution of members of this group observed in eastern and southern regions of Africa where several member species were collected indoors. For example, *An. parensis*, *An. leesoni* and *An. rivulorum* were found in higher densities indoors in a study in Uganda and southern Africa ^37– 41^.

The dominance of *An. funestus* s.s. within the *An*. *funestus* group in this locality further confirms the extremely anthropophilic and endophilic nature of this species which is highly involved in the transmission of human malaria. This result supports the broad geographical distribution of *An*. *funestus* s.s. in Cameroon where it stands as a major malaria vector ^11^. Nonetheless, further studies are required to determine the blood meal source of the major *Anopheles* vectors and their species abundance in outdoor settings in order to have an overview of the vectorial capacity and malaria transmission dynamics in this locality.

Regarding the *An. gambiae* complex, *An. gambiae* was the only species found. This result is similar to previous studies demonstrating that *An. gambiae* s.s. was the major species in rural and semi-rural areas of the Centre and Littoral regions in Cameroon, particularly in Yaounde and Douala ^42– 44^.

### Roles of both vectors in malaria transmission in forested areas

This study confirms the role of *An. funestus* s.s. and *An. gambiae* s.s. in malaria transmission in this locality with sporozoite infection rate of 6% and 5% respectively. This result is similar to *An. funestus* s.s. sporozoite infectivity rate in Mibellon (5%) but higher than in Obout (3.2%) and Tibati (2.94%). Due to the low number of the field-collected *An. gambiae* s.s. during the study period, infection with *Plasmodium* (5%) was similar to *An. funestus* s.s. (6%). Also, this rate is similar to previous results in Cameroon ^10^. Because the location of Elende is close to the Nsimalen-International Airport and to the city of Yaoundé, efforts should be made to reduce the malaria transmission in this locality to avoid it constitute a reservoir for transmission in the city particularly as it was recently shown that mosquitoes can fly over long distances ^12^.

### Multiple and high insecticide resistance in both major vectors constitutes a barrier for vector control in forested areas

Insecticide resistance profile of *An. funestus* s.s. in Elende locality is similar to previous studies in Cameroon documented for this species, where resistance against all pyrethroids and full susceptibility to organophosphates was observed ^11^. The multiple insecticide resistance patterns observed in the *An. funestus* s.s. population to pyrethroids and DDT in Elende corresponds to the trends observed in Gounougou (2012) ^9^ and higher than in Obout (2016) ^45^ but lower than in Tibati (2018) ^35^. Moreover, the high resistance pattern of *An. funestus* s.s. to pyrethroids observed in this locality is similar to that observed in Mibellon (2017) ^11^. This result brings to attention the fact that resistance in *An. funestus* s.s. is pervasive in Cameroon and constitute a threat for operational insecticide-based vector control tools directed at this species. In Cameroon, the massive deployment of LLINs implemented by the Cameroonian Government in the past years has likely contributed to a rapid rise of pyrethroid resistance in *An. funestus* s.s. vector. Moreover, Elende is also located in an area where farming is widely practiced, and agricultural application of pesticides for crop protection apparently imposes a selective pressure that further pilots the increase in resistance level. Similarly, this same pattern of high pyrethroid resistance in *An. funestus* s.s. was observed in Southern Africa in Malawi ^46^ and Mozambique ^47^; the East African region including Uganda ^48^; and West Africa in Ghana ^49^, Benin ^36^ and Nigeria ^50^.

The full reversal to susceptibility observed after PBO exposure to permethrin and deltamethrin, implies that cytochrome P450 genes are playing a notable role in the resistance mechanisms. This increasingly higher resistance to pyrethroids poses a remarkable challenge for malaria control programs in Cameroon and necessitates the urgent implementation of insecticide resistance management strategies so as to prevent failure of future programs directed at scaling-up distribution campaigns of pyrethroid impregnated LLINs.

 Extremely high levels of resistance to several classes of insecticides, including organochlorine, pyrethroid and carbamate, were also noticed in the *An. gambiae* s.s. population from Elende. Moreover, the intense resistance of *An. gambiae* s.s. to 5x and 10x concentration of permethrin and deltamethrin each suggests that the resistance is elevated in this population. This elevated resistance in *An. gambiae* s.s. corresponds with the high level of resistance reported in this species across various sites in Cameroon ^6– 8, 35^. Furthermore, the reduced susceptibility observed against the organophosphates (malathion, fenithrothion and pirimiphos-methyl) in *An. gambiae* s.s. is an indication of possible cross resistance with the carbamates since both insecticides class act on the same nervous system target site. In this regard, carbamates insecticide should be excluded as a replacement to pyrethroid for IRS as this will further select the spread of the resistant allele within the species population of this locality.

 The resistance in *An. gambiae* s.s. was higher compared to *An. funestus* s.s. for almost all the insecticides during this time interval, suggesting a substantial selection pressure acting on *An. gambiae* s.s. This could be as a result of environmental and genetic selection of resistance from breeding sites polluted with pesticides used for crop protection. Since *An. gambiae* s.s. temporal breeding sites are often located nearby areas of crop cultivation, the selection would be enormous in this species compared to *An. funestus* s.s. However, the sample size of field collected *An. gambiae* that laid eggs was low (n=20) and this may have limited the ability to capture the full susceptibility profile of this population to insecticides. Nevertheless, the consistency of the resistance profile with notably the fixation of kdr and RDL in these mosquito population supports the findings that the profile presented in this study could reflect the ongoing resistance pattern.

### Bio efficacy of LLINs in cone assays

Freely distributed LLINs by the National Malaria Contol Programme (NMCP) constitute the central malaria vector control intervention in Elende. The dramatic drop in potency of these solely impregnated pyrethroid nets is comparable to cases reported in other localities in Cameroon ^11^ and Africa ^36, 47^. Resistance to pyrethroids in this species is linked with a marked decline in efficacy to all pyrethroid only LLINs as demonstrated by the diminishing mortality rates against PermaNet 2.0 (<10%) and Olyset net (<25%). Conversely, PBO-based nets demonstrated a greater efficacy with the highest reported by both PermaNet 3.0 top and Olyset plus scoring 100% mortality. This indicates that cytochrome P450 genes are probably propelling pyrethroid resistance in this locality. The higher mortality rate observed with PBO-based nets suggest that these synergist nets including Olyset Plus and PermaNet 3.0 (roof) should be regarded as a substitution to pyrethroid-only nets in areas of increasing resistance fueled by metabolic mechanisms particularly for cytochrome P450s as it is the situation for *An*. *funestus* s.s. ^29^.

### Elevated metabolic resistance in *An. funestus* differs with high levels of knockdown resistance in *An. gambiae*

The full susceptibility noticed for pyrethroids in *An. funestus* s.s. after first exposure to PBO points out that metabolic resistance mediated by cytochrome P450s is the main mechanism ^51^. This is linked to previous studies which confirm the absence of *kdr* target site sensitivity mutation in this species in Cameroon ^9^ and across Africa ^52^. In the absence of voltage-gated sodium channel knockdown resistance mutations in *An. funestus* s.s. ^52^, this study demonstrated that pyrethroid resistance in Elende populations of *An. funestus* s.s. is possibly steered by metabolic resistance machinery. Overall, the role of metabolic resistance is apparent by the marked up-regulation of the three P450 genes ( *CYP6P5*, *CYP6P9a* and *CYP6P9b*) already reported as essential genes conferring pyrethroid resistance in *An. funestus* s.s. populations across Africa ^30^.

The absence of the *CYP6P9a* resistance allele in *An. funestus* s.s. population from Elende corresponds to the study by Weedall *et al.* ^26^. This confirms the fact that this mutation, fixed in mosquitoes from southern Africa is not yet present in mosquitoes from Central/West Africa ^26^.

Cross-resistance to DDT and pyrethroids has been demonstrated to be conferred by GSTe2. In relation to this, the frequency of the L119F-GSTe2-resistant allele in the Elende field population (48%) is higher than in Mibellon (28%), Tibati (10.2%) and lower than in Gounougou (52%). Similarly, across Africa, the frequency of the DDT resistance marker was closer to that observed in Democratic Republic of Congo ^53^ and Ghana ^49^; higher than the frequency reported in eastern part of Africa, Uganda ^37, 38^ although lower to studies in Benin ^36^.

The frequency of the 296S-RDL-resistant allele is 100%, which is higher than in the northern region, particularly in Mibellon (9.7%), Gounougou (14.6%) and Tibati (0.4%). However, this result is similar to mortality rate recorded in *An. funestus* s.s. from Obout that exhibited very high level of resistance to dieldrin (4.35% mortality rate) ^45^. This high frequency could be as a result of strong resistance selection due to environmental persistence of insecticide residues since its withdrawal from public and agricultural use in Cameroon.

The elevated resistance levels to pyrethroids in *An. gambiae* s.s. accords with the increased frequency of the 1014F *kdr* allele (94.9%). This correlates with past studies done in Africa where high pyrethroid resistance in *An. gambiae* s.l. has been coupled with almost fixed *kdr* allele in the population, as recently reported in DR Congo ^53^, or earlier in Côte d’Ivoire ^54^. Consistent with previous research performed in other parts of Cameroon ^8, 35^, this study found elevated frequencies of the *kdr* mutation in An *. gambiae* s.s. population in Elende that has almost reached fixation.

The very low frequency of the 1014S *kdr* allele in Elende (1.28%) is in parallel to earlier reports across Cameroon exhibiting that this target site resistance mutation, originally discovered in East Africa, is gradually spreading to Central and West Africa although still at very low occurrence ^55^.

The presence of the 119S *ace*- *1* mutation in *An. gambiae* s.s. population is in line with the reduced susceptibility observed in this population to carbamates and organophosphates ^34, 56, 57^. The use of carbamates and organophosphates may be regarded as an alternative for the management of this highly insecticide resistant vector population although the detection of *Ace*- *1* is also a cause of concern.

Similar studies involving large sample sizes should be conducted across different ecological settings in Cameroon to establish the epidemiological and entomological parameters of malaria transmission and investigate the resistance profile of malaria vectors to existing insecticides. Specifically, a longitudinal survey would obviously provide useful and interesting information on the seasonal species composition and abundance, in-depth knowledge on the biology of each species, mosquito host-seeking and resting preferences, pattern of insecticide resistance, frequency of insecticide resistance genes and the role played by *Anopheles* vectors in malaria transmission in this locality over time. Data produced from such future studies will be relevant in generating additional significant information required to strengthen malaria control.

## Conclusion

This study reports the preliminary characterization and resistance profile of endophilic malaria vectors *An. funestus* and *An. gambiae* in Elende locality, situated close to a port of entry in Yaounde, the capital city of Cameroon. The significant *Plasmodium* sporozoite infection rate alongside the resistance to multiple insecticide classes observed in these vector populations highlights the challenges that public health vector control programs encounter in sustaining the regular effectiveness of contemporary insecticide-based control interventions aimed at reducing malaria transmission in forested areas. More particularly, the baseline resistance observed against the carbamates and possible resistance against the organophosphates constitutes a major concern for IRS; while suggesting the susceptibility evaluation of *Anopheles* malaria vectors in this locality to neonicotinoids and pyrrole insecticides in preparation for indoor residual spraying campaigns with novel insecticide ingredients. Also, this study further provides operational evidence to National Malaria Control Programs for a shift from mass distribution of pyrethroid-only LLINs to second-generation bed nets (containing synergist) in areas where high resistance is driven by metabolic mechanisms notably cytochrome P450s.

## Data availability

### Underlying data

Open Science Framework: Elevated Plasmodium sporozoite infection and multiple insecticide resistance in the principal malaria vectors Anopheles funestus and Anopheles gambiae in a forested locality close to the airport of Yaoundé, the Capital city of Cameroon. https://doi.org/10.17605/OSF.IO/XN68J ^34^.

This project contains the following underlying data:

- 1-Raw output Ct values_qRT-PCR experiment_data_including housekeeping genes (XLSX). (Raw Ct values generated during qRT-PCR experiments.)
- 2-Taqman derived_ *Plasmodium* infection rates_ *An. funestus* and *An. gambiae*_Elende (XLSX). ( *Plasmodium* mosquito infection rates, measured via TaqMan assay.)
- 3- *An. funestus* and *An. gambiae*_PCR species identification (XLSX). (Mosquito species identification data, performed using PCR.)
- 4-Pyrethroid exposure experiments & WHO insecticide susceptibility & PBO synergist assay (XLSX). (Raw insecticide susceptibility mortality data.)
- 5-Data generated from cone assay (XLSX). (Raw mortality data generated via cone assays.)
- 6-Data generated for resistance marker genotyping experiments (XLSX). (Raw qRT-PCR resistance genotyping data.)

The *An. funestus* s.s. ITS2 nucleotide sequences have been deposited in GenBank with accession numbers: MT991011 to MT991016.

Data are available under the terms of the Creative Commons Zero "No rights reserved" data waiver (CC0 1.0 Public domain dedication).
