# Supplementary material for: Multiple insecticide resistance and Plasmodium infection in the principal malaria vectors Anopheles funestus and Anopheles gambiae in a forested locality close to the Yaoundé airport, Cameroon
Source: Wellcome Open Res. 2020 Nov 5;5:146. Originally published 2020 Jun 23. [Version 2] doi: 10.12688/wellcomeopenres.15818.2 (PMC7667521; doi:10.12688/wellcomeopenres.15818.2)
Supplement: Peer review history

# Peer review: Reviewer response for version 2

**DOI:** 10.21956/wellcomeopenres.17917.r41288

**Published:** 2020-11-13

**Authors:** Louisa A. Messenger

**Affiliations:** 1 Department of Disease Control, Faculty of Infectious Tropical Diseases, London School of Hygiene and Tropical Medicine, London, UK

Many thanks to the authors for addressing all of my comments and concerns.

Is the work clearly and accurately presented and does it cite the current literature?

Yes

If applicable, is the statistical analysis and its interpretation appropriate?

Not applicable

Are all the source data underlying the results available to ensure full reproducibility?

No

Is the study design appropriate and is the work technically sound?

Partly

Are the conclusions drawn adequately supported by the results?

Partly

Are sufficient details of methods and analysis provided to allow replication by others?

Yes

Reviewer Expertise:

Medical entomology, population genetics, insecticide resistance, molecular biology

I confirm that I have read this submission and believe that I have an appropriate level of expertise to confirm that it is of an acceptable scientific standard.

# Peer review: Reviewer response for version 1

**DOI:** 10.21956/wellcomeopenres.17349.r39209

**Published:** 2020-08-18

**Authors:** Mamadou Ousmane Ndiath, Jessy Goupeyou-Youmsi

**Affiliations:** 1 Disease Control & Elimination Theme, Medical Research Council Unit, London School of Hygiene and Tropical Medicine, Fajara, The Gambia; 2 MAC Communicable Diseases Action Center, , College of Medicine - University of Malawi, Blantyre, Malawi

**General comments**

 The authors characterized the species composition of *Anopheles* mosquitoes at Elende in Cameroon, investigated the insecticide resistance profile (using the four classes of insecticides) of two mosquito species ( *An. funestus* s.s. and *An. gambiae* s.s) that are major malaria vectors in Cameroon, and evaluated the sporozoite carriage rate in these two mosquito species. This research study is relevant to several fields including entomology and malaria transmission. The results of this study will help strengthen current vector control interventions for malaria control in Cameroon.  The authors reviewed the existing literature adequately and provided the source data underlying the results available to ensure full reproducibility. However, the study design is partly appropriate, leading to some conclusions which are not adequately supported by the results. See major and minor comments below.

- **Major comments**: The sample size is low, both for the insecticide susceptibility test and molecular biology. Based on the underlying data raw, only one replicate was done for the PBO synergist assays, while according to the WHO test procedures for insecticide resistance monitoring in malaria vector mosquitoes, this should be at least 2 replicates (not to be confused with the number of test tubes per treatment).
- **Minor comments**: Indoor collection of mosquitoes only does not allow to characterize mosquito species composition. The good point is that the authors highlighted this limitation in the discussion. We suggest the authors to remove this as an objective since the goal was to collect bloodfed mosquitoes to produce the F1 progeny for insecticide resistance assays. We also suggest the authors to rephrase the expression “high *Plasmodium* infection rate” throughout the manuscript for the reason we gave in the following paragraph.

**Title**

- We suggest authors to remove the word “Elevated” in the title and to rephrase it, since the results of *Plasmodium* infection rate are not strong enough to support the affirmation “Elevated *Plasmodium* sporozoite infection” *. *Indeed, (1) the sample size is very low (150 for *An. funestus* and 39 for *An gambiae*), (2) mosquitoes analyzed were bloodfed (=> more chance of contamination) and (3) the PCR assay was not *Plasmodium* stage-specific.

**Abstract**

- It will help the reader to specify at the Methods section the insecticides used or at least that the four classes of insecticides were used.
- Add the word “THE” in the following sentence: “ *Anopheles funestus* s.s. was THE most…”.
- It would be good to use either the name of the insecticide or the insecticide class when giving results.
- The term “high *Plasmodium* sporozoite rate” is a very strong statement given the results.

**Introduction**

- In the sentence “…the insecticide resistance profiles of *An. funestus* s.s. and *An. gambiae* s.s. population collected…”, add S to the word population. 

**Methods**

- WHO insecticide susceptibility test: clarify why the resistance intensity to pyrethroids was studied only for *An. gambiae.*
- PBO synergist assay:
  - How many replicates were done?
  - In the sentence “…exposure to permethrin (0.75%) and deltamethrin (0.05%) for another 1 h interval.”, replace “and” by OR and “interval” by EXPOSURE. 
- Assessment of bed net efficacy using cone assay: “Due to the insufficiency of *Anopheles gambiae* s.s.,…”, replace “insufficiency by LOW ABUNDANCE. 

**Results**

*Plasmodium* infection rate:

- About the TaqMan Assay, what was the number of cycles for denaturation? (give the PCR conditions if different from what of Bass et al, 2008). This an important parameter to take into account as that will help to choose the Ct beyond which all amplified samples will be considered as negative. Looking at the underlying data raw, all samples with amplification signal after the 39 ^th^ cycle should have been considered as negative. The Ct value 37.39 of the positive control OVM is quite high (replicate 59 in tab 2).
- In the underlying data raw, we counted 9 (not 10) *An. funestus* samples positive to *Plasmodium* (7 *P. falciparum* and 2 OVM) *. *Please check again.
- About the sentence “The *Plasmodium* infected mosquitoes revealed by TaqMan was further confirmed with nested PCR.”, rephrase it like this: *Plasmodium* infected mosquitoes revealed by TaqMan were further tested with nested PCR.
- Insecticide susceptibility assays: Indicate the total number of mosquitoes tested per treatment (insecticide) for both *An. funestus *and *An. gambiae*. Also add this information in the figure 2.
- PBO synergist assays
  - How many replicates were done?
  - How many mosquitoes tested per treatment? Indicate this in the figure 3.
- In the underlying data raw (WHO susceptibility test and cone bioassays), check again start and end times so that it matches 1H.

**Discussion**

- Taking into account the information given by the authors about the study area, it seems like Elende suits better a peri-urban setting.
- “Due to the low number of the field-collected *An. gambiae* s.s. during the study period, infection with *Plasmodium* (5%) was lower compared to *An. funestus* s.s. (6.6%).”: the authors should support this statement with statistical analysis.

Is the work clearly and accurately presented and does it cite the current literature?

Yes

If applicable, is the statistical analysis and its interpretation appropriate?

Yes

Are all the source data underlying the results available to ensure full reproducibility?

Yes

Is the study design appropriate and is the work technically sound?

Partly

Are the conclusions drawn adequately supported by the results?

Partly

Are sufficient details of methods and analysis provided to allow replication by others?

Yes

Reviewer Expertise:

NA

We confirm that we have read this submission and believe that we have an appropriate level of expertise to confirm that it is of an acceptable scientific standard.

# Response

**Published:** 2020-10-07

**Authors:** Nkemngo Francis Nongley

**Affiliations:** Centre for Research in Infectious Diseases, Cameroon

**Mamadou Ousmane Ndiath**

 Disease Control & Elimination Theme, Medical Research Council Unit, London School of Hygiene and Tropical Medicine, Fajara, The Gambia

**Jessy Goupeyou-Youmsi**

 MAC Communicable Diseases Action Center, , College of Medicine - University of Malawi, Blantyre, Malawi

**General comments**

 The authors characterized the species composition of Anopheles mosquitoes at Elende in

 Cameroon, investigated the insecticide resistance profile (using the four classes of insecticides) of two mosquito species ( *An. funestus s.s. *and *An. gambiae s.s*) that are major malaria vectors in

 Cameroon, and evaluated the sporozoite carriage rate in these two mosquito species. This

 research study is relevant to several fields including entomology and malaria transmission. The

 results of this study will help strengthen current vector control interventions for malaria control in Cameroon. The authors reviewed the existing literature adequately and provided the source data underlying the results available to ensure full reproducibility. However, the study design is partly appropriate, leading to some conclusions which are not adequately supported by the results. See major and minor comments below.

**Comment 1:**

**Major comments:** The sample size is low, both for the insecticide susceptibility test and

 molecular biology. Based on the underlying data raw, only one replicate was done for the

 PBO synergist assays, while according to the WHO test procedures for insecticide resistance

 monitoring in malaria vector mosquitoes, this should be at least 2 replicates (not to be

 confused with the number of test tubes per treatment).

**Response: **

- We thank the reviewer for this comment. We are sorry if the explanation of the samples used was not clear enough. However, we will like to emphasize that for all tests done for *An. funestus*, we performed 4 replicates with around 80-100 mosquitoes in total, and for PBO synergist assays we did the same which is enough to capture the susceptibility profile in this population. This is now better explained in the text notably for PBO wherein a total of 489 mosquitoes were in fact used including the controls as we also indicated a similar response to a query from the other reviewer.

**Comment 2:**

 Minor comments: Indoor collection of mosquitoes only does not allow to characterize

 mosquito species composition. The good point is that the authors highlighted this limitation

 in the discussion. We suggest the authors to remove this as an objective since the goal was

 to collect blood-fed mosquitoes to produce the F1 progeny for insecticide resistance assays.

**Response:**

- We agree indoor collection does not substantially allow for characterization of mosquito species composition; but it does provide a basis for identifying the endophilic vectors which constitute a great arm for indoor malaria transmission. We have revised this objective as highlighted by the authors in the paragraph before the method section as: This included the characterization of the **endophilic mosquito species** composition, investigation of the insecticide resistance profiles of *An. funestus *s.s. and *An. gambiae *s.s. populations collected in Elende in 2019.

**Comment 3:**

 We also suggest the authors to rephrase the expression “high *Plasmodium* infection rate”

 throughout the manuscript for the reason we gave in the following paragraph.

**Response:**

- We have now removed high as suggested by the reviewer.

**Comment 4:**

 ○Title: We suggest authors to remove the word “Elevated” in the title and to rephrase it, since the results of *Plasmodium* infection rate are not strong enough to support the affirmation

 “Elevated Plasmodium sporozoite infection”. Indeed, (1) the sample size is very low (150 for

*An. funestus* and 39 for *An gambiae*), (2) mosquitoes analyzed were bloodfed (=> more

 chance of contamination) and (3) the PCR assay was not Plasmodium stage-specific.

**Response: **

- The title has been revised accordingly and is as follows “Multiple insecticide resistance and *Plasmodium* infection in the principal malaria vectors *Anopheles funestus* and *Anopheles gambiae* in a forested locality close to the Yaoundé airport, Cameroon”

**Comment 5:**

 ○ Abstract: It will help the reader to specify at the Methods section the insecticides used or at least that the four classes of insecticides were used.

**Response: **

- We thank the reviewer for this comment. We have revised the sentence to include the fact that the four classes of insecticides were used. Thus, the sentence has been modified to “Bioassays were performed to assess resistance profile to the four insecticides classes” 

**Comment 6:**

 ○ Add the word “THE” in the following sentence: “ *Anopheles funestus s.s*. was THE most…”.

 It would be good to use either the name of the insecticide or the insecticide class when

 giving results.

**Response: **

- We sincerely thank the reviewer for this detail comments. “The” has been added to the sentence and the various pyrethroid insecticides used have been included in the sentence which now reads “Both species exhibited high levels of resistance to the pyrethroids, permethrin and deltamethrin (<40% mortality)”.

**Comment 7:**

 ○ The term “high *Plasmodium* sporozoite rate” is a very strong statement given the results.

**Response: **We agree and high has been removed.

**Comment 8:**

 Introduction

 In the sentence “…the insecticide resistance profiles of *An. funestus s.s.* and *An. gambiae s.s.*

 population collected…”, add S to the word population.

**Response: **

- Thank you for the comment.  “S” has been added.

**Comment 9:**

 ○Methods

 WHO insecticide susceptibility test: clarify why the resistance intensity to pyrethroids was

 studied only for *An. gambiae*.

**Response: **

- We did not have sufficient numbers of F1 *An. funestus* progeny to do the resistance intensity bioassay. We plan to perform intensity bioassay on An. funestus in future studies.

**Comment 10:**

 ○ PBO synergist assay:

 ○ How many replicates were done?

**Response: **

- The following replicates were conducted for PBO synergist assay:

Permethrin 0.75%: 04 replicates

 PBO 4% + Permethrin 0.75%: 04 replicates

 Deltamethrin 0.05%: 04 replicates

 PBO 4% + Deltamethrin 0.05%: 04 replicates

 PB0 4%: 03 replicates

 Control: 03 replicate

**Comment 11:**

 In the sentence “…exposure to permethrin (0.75%) and deltamethrin (0.05%) for

 another 1 h interval.”, replace “and” by OR and “interval” by EXPOSURE.

**Response:** Thanks for this comment. These changes have been made.

**Comment 12:**

 ○Assessment of bed net efficacy using cone assay: “Due to the insufficiency of Anopheles

*gambiae *s.s.,…”, replace “insufficiency by LOW ABUNDANCE.

**Response: **

- Thank you. This change has been made.

**Comment 13:**

 ○Results

*Plasmodium* infection rate:

 About the TaqMan Assay, what was the number of cycles for denaturation? (give the PCR

 conditions if different from what of Bass et al, 2008). This an important parameter to take

 into account as that will help to choose the Ct beyond which all amplified samples will be

 considered as negative. Looking at the underlying data raw, all samples with amplification

 signal after the 39th cycle should have been considered as negative. The Ct value 37.39 of

 the positive control OVM is quite high (replicate 59 in tab 2).

**Response: **

- Basically, the PCR conditions were: segment 1 [1 cycle at 95 ^0^C/10mins] and segment 2 [40 cycles at 92 ^0^C/15s and 60 ^0^C/1min]. We thank the reviewer for bringing this point up. The Ct (cycle threshold) is defined as the number of cycles required for the fluorescent signal to cross the threshold (i.e. exceeds background level). As such, high Ct values can also mean lower (and even too little) amounts of the target nucleic acid and not necessarily a negative sample. Indeed, the high Ct value of the P. OVM positive control indicates that the *Plasmodium* parasite DNA was present in little amount. This P. OVM control was further confirmed with nested PCR where a band size of 144bp was observed indicative of *P. malariae*.

 

**Comment 14:**

 ○In the underlying data raw, we counted 9 (not 10) *An. funestus* samples positive to

*Plasmodium* (7 P *. falciparum* and 2 OVM). Please check again.

**Response: **

- We sincerely appreciate this detail comment from the reviewer. We double-checked again from the original data and agree with your remark. In line, the *Plasmodium* infection rate for *An. funestus* was 6% (09/150). We have corrected the infection rate and associated statistics in the revised version of the manuscript.

**Comment 15:**

 ○About the sentence “The Plasmodium infected mosquitoes revealed by TaqMan was further

 confirmed with nested PCR.”, rephrase it like this: *Plasmodium* infected mosquitoes revealed

 by TaqMan were further tested with nested PCR.

**Response: **

- We thank the reviewer for noting this. As mentioned, the sentence has been rephrased to: “Plasmodium infected mosquitoes revealed by TaqMan were further tested with nested PCR” in the revised version of the manuscript.

**Comment 16:**

 ○ Insecticide susceptibility assays: Indicate the total number of mosquitoes tested per

 treatment (insecticide) for both *An. funestus* and *An. gambiae*. Also add this information in

 PBO synergist assays

 ○ How many replicates were done?

 ○ How many mosquitoes tested per treatment? Indicate this in the figure 3.

 ○ In the underlying data raw (WHO susceptibility test and cone bioassays), check again start

 and end times so that it matches 1H.

**Response: **

- Thank you for this remark. The total number of mosquitoes tested per insecticide for both vectors have been included in the revised version of the manuscript. Similarly, the same revision has been done for the PBO assay in the text displaying fig 3. Also, the underlying raw data has been re-checked.

**Comment 17:**

 ○Discussion

 Taking into account the information given by the authors about the study area, it seems like

 Elende suits better a peri-urban setting.

**Response: **

- We agree with the reviewer. We have modified the suggestion accordingly.

**Comment 18:**

 ○ “Due to the low number of the field-collected *An. gambiae s.s*. during the study period,

 infection with *Plasmodium* (5%) was lower compared to *An. funestus s.s.* (6.6%).”: the authors

 should support this statement with statistical analysis

**Response: **

- Thank you for this comment for which we agree. We have modified the sentence of this comparison.

# Peer review: Reviewer response for version 1

**DOI:** 10.21956/wellcomeopenres.17349.r39478

**Published:** 2020-07-23

**Authors:** Louisa A. Messenger

**Affiliations:** 1 Department of Disease Control, Faculty of Infectious Tropical Diseases, London School of Hygiene and Tropical Medicine, London, UK

This article characterizes malaria vector dynamics (species composition, insecticide resistance phenotypic profiles and mechanisms and Plasmodium infection) in a forested region of Cameroon, where little previously data have been reported previously. This article provides information to guide local prospective malaria vector control measures and resistance management strategies.

 My main concern with this study is the very small number of mosquitoes. The mosquito collections were only performed for two days – this is a very short period of time. Indoor mosquito collections can certainly be more difficult and not as productive as other sampling methods, but the small sample sizes in this study do limit the conclusions the authors can make considerably.

 Minor comments:

- In the methods, “study area and mosquito collection” section, instead of speculating that the marshy lands are “accompanied by an increased malaria transmission”, can you please provide some data to support this?
- In the methods, “study area and mosquito collection” section, how many houses were sampled for mosquito collections to give an idea of whether you likely just sampled mosquito siblings or were able to gather more-like population-level data for this area.
- In the methods “study area and mosquito collection” section, can you specify which pyrethroid LLINs have been distributed, to enable better interpretation of the resistance data.
- In the methods “WHO insecticide susceptibility bioassays” section, why was DDT and fenitrothion tested? I just wonder if it might have been a better use of your mosquitoes to test *An. funestus* s.l. with pirimiphos-methyl as well, given it is a potential IRS candidate in Cameroon.
- In the methods “WHO insecticide susceptibility bioassays” section, did you interpret the 5X and 10X diagnostic results with reference to the latest WHO guidelines? I.e. at 5X <98% mortality indicates moderate to high intensity resistance and at 10X <98% mortality indicates high intensity resistance.
- In methods “molecular species identification” section, have the authors deposited the ITS2 sequences in GenBank, or another open-access data repository (I could not easily find it among the supporting data files)? If not, can they please do so? It would be interesting to see if there was some sequence variability which might explain the proportion of non-amplifying *An. funestus* s.s. using the cocktail PCR.
- In the genotyping methods sections, why was genotyping performed on F0 mosquitoes, when to directly relate individual mosquito phenotype to its genotype it should have been performed with F1 mosquitoes? This was done with the transcription profiling. Is this a simple typing mistake?
- In the “transcription profiling of candidate resistance associated genes”, what do you mean by “F1 females each that recovered after 1 h exposure”? Do you mean mosquitoes which survived the 1 hr exposure and can be classified as “resistant” (as you do subsequently) or do you mean those that were knocked-down during exposure and then later revived after the test? As these are two distinct phenotypic populations, please clarify.
- In the results “species identification” section, the mosquito populations under evaluation, with founding population sizes of 196 *An. funestus* s.s. and 20 *An. gambiae* s.s., are likely to be very genetically restricted/highly related, which limits the applicability and usefulness of these data. Similarly, the number of mosquitoes screened for malaria and genotyped for resistance mutations was really low. This point is touched upon in the discussion but it is quite likely that this study is biased by familial isolation effects.
- In the results “insecticide susceptibility assays” or in the figures, can the authors please specify the number of mosquitoes tested per dose? According to the WHO guidelines this should be approximately 100 mosquitoes per dose (plus appropriate controls), but the authors report screening 598 mosquitoes for 7 insecticides, so I assume less replicates were performed? Especially for PBO – I am not sure how you managed to run negative and positive controls, PBO pre-exposures and pyrethroid-only exposures in parallel, using only 169 mosquitoes?
- Given the number of experimental limitations, including sample sizes and collection duration, it would be good to see the authors acknowledge these more openly in the discussion please.

Is the work clearly and accurately presented and does it cite the current literature?

Yes

If applicable, is the statistical analysis and its interpretation appropriate?

Not applicable

Are all the source data underlying the results available to ensure full reproducibility?

No

Is the study design appropriate and is the work technically sound?

Partly

Are the conclusions drawn adequately supported by the results?

Partly

Are sufficient details of methods and analysis provided to allow replication by others?

Yes

Reviewer Expertise:

Medical entomology, population genetics, insecticide resistance, molecular biology

I confirm that I have read this submission and believe that I have an appropriate level of expertise to confirm that it is of an acceptable scientific standard, however I have significant reservations, as outlined above.

# Response

**Published:** 2020-10-07

**Authors:** Nkemngo Francis Nongley

**Affiliations:** Centre for Research in Infectious Diseases, Cameroon

**Louisa A. Messenger**

**Department of Disease Control, Faculty of Infectious Tropical Diseases, London School of Hygiene and Tropical Medicine, London, UK**

 This article characterizes malaria vector dynamics (species composition, insecticide resistance phenotypic profiles and mechanisms and Plasmodium infection) in a forested region of Cameroon, where little previously data have been reported previously. This article provides information to guide local prospective malaria vector control measures and resistance management strategies.

**Query 1:**

 My main concern with this study is the very small number of mosquitoes. The mosquito collections were only performed for two days – this is a very short period of time. Indoor mosquito collections can certainly be more difficult than and not as productive as other sampling methods, but the small sample sizes in this study do limit the conclusions the authors can make considerably.

**Response:**

- We thank the reviewer for this comment. A total of 196 randomly collected *An. funestus* laid eggs. If we considered that it has been previously shown that the average number of mosquitoes that constitute a population (a deme) is around 50 individuals (Besansky et al 1997), the number of 196 might be sufficient to capture the overall susceptibility profile of this population. For *An. gambiae* a total of 20 did lay eggs and we agree that this is low, but this is the maximum we could obtain. This limitation for *An. gambiae* is now highlighted in the manuscript as follows: “However, the sample size of field collected *An. gambiae* that laid eggs was low (n=20) and this may have limited the ability to capture the full susceptibility profile of this population to insecticides. Nevertheless, the consistency of the resistance profile with notably the near fixation kdr and that of RDL in these mosquito population supports the findings that the profile presented in this study could reflect the ongoing resistance pattern”.

 

**Query 2:**

 Minor comments:

 In the methods, “study area and mosquito collection” section, instead of speculating that the

 marshy lands are “accompanied by an increased malaria transmission”, can you please provide some data to support this?

**Response:**

- We agree with the reviewer that no data is currently available to support the increase malaria transmission in Elende due to the marshy lands. Therefore, we have deleted the related statement: “accompanied by an increase malaria transmission”.  

**Query 3:**

 In the methods, “study area and mosquito collection” section, how many houses were sampled for mosquito collections to give an idea of whether you likely just sampled mosquito siblings or were able to gather more-like population-level data for this area.

**Response:**

- The number of houses sampled for mosquito collection has been included in the text as follows: “from 15-20 randomly selected houses to avoid sampling individuals from single female egg batches and to obtain a representative population-level data for this area”.

**Query 4:**

 In the methods “study area and mosquito collection” section, can you specify which pyrethroid LLINs have been distributed, to enable better interpretation of the resistance data.

**Response:**

- Thank you for this interesting remark. PermaNet 2.0 (Deltamethrin 55mg/m ^2^) was the pyrethroid LLIN distributed in this locality.

**Query 5:**

 In the methods “WHO insecticide susceptibility bioassays” section, why was DDT and fenitrothion tested? I just wonder if it might have been a better use of your mosquitoes to test An. funestus s.l. with pirimiphos-methyl as well, given it is a potential IRS candidate in Cameroon.

**Response: **

- We agree that testing pirimiphos-methyl would have been ideal. Unfortunately, this insecticide paper was not available. This will surely be done in the future. We tested DDT and fenitrothion to obtained a profile of resistance against all 4 classes of insecticides.

**Query 6:**

 In the methods “WHO insecticide susceptibility bioassays” section, did you interpret the 5X and 10X diagnostic results with reference to the latest WHO guidelines? I.e. at 5X <98% mortality indicates moderate to high intensity resistance and at 10X <98% mortality indicates high intensity resistance.

**Response: **

- We thank the reviewer for this comment. We have now clearly indicated that the WHO criteria was used to assess the resistance intensity as shown in the sentence below in the text. “High intensity resistance to 5x and 10x permethrin was observed with a recorded mortality rate of 31.74% and 44.30% respectively which according to the WHO guidelines (WHO, 2016) indicates a high intensity of resistance in Elende.”

**Query 7:**

 In methods “molecular species identification” section, have the authors deposited the ITS2

 sequences in GenBank, or another open-access data repository (I could not easily find it

 among the supporting data files)? If not, can they please do so? It would be interesting to see if there was some sequence variability which might explain the proportion of non-amplifying An. *funestus *s.s. using the cocktail PCR.

**Response: **

- Thank you for this observation. The ITS2 sequences have been deposited in GenBank with accession numbers: MT991011 to MT991016. These have been included in the text.

**Query 8:**

 In the genotyping methods sections, why was genotyping performed on F0 mosquitoes, when to directly relate individual mosquito phenotype to its genotype it should have been performed with F1 mosquitoes? This was done with the transcription profiling. Is this a simple typing mistake?

**Response: **

- Genotyping was done for the F0 mosquitoes to obtain the frequency of resistance in the field since they are the operationally relevant exposed population.
- The transcriptional profiling was done of F1 as it is easier to get enough numbers as three pool of 30 mosquitoes alive after performing the bioassay. Also controlling the age of the mosquitoes so as to perform bioassay with mosquitoes aged 3 to 5 days old is complicated with F0 for *An. funestus *as finding their larvae is difficult.

**Query 9:**

 In the “transcription profiling of candidate resistance associated genes”, what do you mean by “F1 females each that recovered after 1 h exposure”? Do you mean mosquitoes which survived the 1 hr exposure and can be classified as “resistant” (as you do subsequently) or do you mean those that were knocked-down during exposure and then later revived after the test? As these are two distinct phenotypic populations, please clarify.

**Response: **

- Thanks for bringing this up. Here, we are referring to the mosquitoes alive 24 hours post 1hour insecticide exposure.

**Query 10:**

 In the results “species identification” section, the mosquito populations under evaluation, with founding population sizes of 196 An. funestus s.s. and 20 An. gambiae s.s., are likely to be very genetically restricted/highly related, which limits the applicability and usefulness of these data.

 Similarly, the number of mosquitoes screened for malaria and genotyped for resistance mutations was really low. This point is touched upon in the discussion but it is quite likely that this study is biased by familial isolation effects.

**Response:  **

- This comment is already addressed above in the major comments section (Query 1). For *An. gambiae*, we highlighted this limitation in the discussion. For *An. funestus* the number of 196 F0 females that laid eggs and thus contributing to the F1 is high enough (based on expected size of a deme) to be representative of the genetic variability in this population and reflects similar studies previously performed on this species for which it is difficult to collect field larvae for such tests.  

**Query 11:**

 In the results “insecticide susceptibility assays” or in the figures, can the authors please specify the number of mosquitoes tested per dose? According to the WHO guidelines this should be approximately 100 mosquitoes per dose (plus appropriate controls), but the authors report screening 598 mosquitoes for 7 insecticides, so I assume less replicates were performed?

**Response: **

- Thank you for this comment. In total, we employed the sum of 638 *An. funestus s.s* F1 for the insecticide susceptibility assay. Unfortunately, due to the low number, we could not perform the test with 100 samples for all insecticides tested. Nevertheless, the results presented provide an indication of the current susceptibility profile. We have included the number of mosquitoes tested per treatment in the manuscript.

**Query 12:**

 Especially for PBO – I am not sure how you managed to run negative and positive controls, PBO pre-exposures and pyrethroid-only exposures in parallel, using only 169 mosquitoes?

**Response: **

- The positive and negative controls were performed for PBO but with less than 100 mosquitoes and this was done at the same time as the other tests to take advantage of a common negative control.
- Thank you for this comment. Overall, a total of 489 F1 female *An. funestus s.s*. mosquitoes were utilized for the complete PBO synergist assay (which includes parallel and simultaneous exposure of the F1 female offspring mosquito population to: pyrethroid only impregnated papers, PBO only impregnated papers, PBO + pyrethroid exposures and controls). In the PBO synergist assay section of the manuscript, 169 F1 *An. funestus s.s.* mosquitoes referred only to the pyrethroid + PBO exposed not including the pyrethroids only exposed and the controls. When these are included, the total is 489.

**Query 13:**

 Given the number of experimental limitations, including sample sizes and collection duration, it would be good to see the authors acknowledge these more openly in the discussion please.

**Response:**

 The limitation in sample size concerns mainly *An. gambiae* which is now acknowledged in the discussion as suggested by the reviewer.
